# Two types of double chambered left ventricle: a case series

**DOI:** 10.1093/ehjcr/ytaf023

**Published:** 2025-01-23

**Authors:** Aijuan Fang, Lei Liu, Hui Chen, Xiuling Ma, Hongming Yu, Jing Yao

**Affiliations:** Department of Ultrasound Medicine, Affiliated Drum Tower Hospital of Nanjing University Medical School, No.321 Zhongshan Road, Nanjing, JS 210000, China; Medical Image Center, Affiliated Drum Tower Hospital of Nanjing University Medical School, No.321 Zhongshan Road, Nanjing, JS 210000, China; Department of Ultrasound Medicine, Affiliated Drum Tower Hospital of Nanjing University Medical School, No.321 Zhongshan Road, Nanjing, JS 210000, China; Medical Image Center, Affiliated Drum Tower Hospital of Nanjing University Medical School, No.321 Zhongshan Road, Nanjing, JS 210000, China; Department of Ultrasound Medicine, Affiliated Drum Tower Hospital of Nanjing University Medical School, No.321 Zhongshan Road, Nanjing, JS 210000, China; Medical Image Center, Affiliated Drum Tower Hospital of Nanjing University Medical School, No.321 Zhongshan Road, Nanjing, JS 210000, China; Department of Ultrasound Medicine, Affiliated Drum Tower Hospital of Nanjing University Medical School, No.321 Zhongshan Road, Nanjing, JS 210000, China; Medical Image Center, Affiliated Drum Tower Hospital of Nanjing University Medical School, No.321 Zhongshan Road, Nanjing, JS 210000, China; Medical Image Center, Affiliated Drum Tower Hospital of Nanjing University Medical School, No.321 Zhongshan Road, Nanjing, JS 210000, China; Department of Ultrasound Medicine, Affiliated Drum Tower Hospital of Nanjing University Medical School, No.321 Zhongshan Road, Nanjing, JS 210000, China; Medical Image Center, Affiliated Drum Tower Hospital of Nanjing University Medical School, No.321 Zhongshan Road, Nanjing, JS 210000, China

**Keywords:** Congenital, Cardiac, Left ventricle, Echocardiography, Case series

## Abstract

**Background:**

Double chambered left ventricle (DCLV) is a rare anatomical variant of the left ventricular structure characterized by the division of the left ventricle into two distinct chambers due to abnormal muscle bundles or septa. This anomaly typically results in primary and secondary chambers within the left ventricle. Due to the lack of conspicuous clinical symptoms, DCLV is often overlooked.

**Case summary:**

The first patient was a 69-year-old male experiencing intermittent chest tightness for several weeks. Transthoracic echocardiography indicated the presence of Type A DCLV, which was subsequently confirmed by 3D echocardiography and cardiac magnetic resonance imaging. An additional case is also worthy of note, during a routine transthoracic echocardiographic examination of a 48-year-old male, we unexpectedly discovered Type B DCLV. His unique cardiac structure was clearly demonstrated through 3D echocardiography.

**Discussion:**

Double chambered left ventricle is a rare structural heart malformation often overlooked due to atypical symptoms. This paper presents two cases of DCLV, illustrating two distinct classifications. Comprehensive understanding of the imaging manifestations of DCLV is imperative for accurate clinical diagnosis.

**Learning points**

- Double chambered left ventricle (DCLV) is defined as a myocardial bundle or septum that occurs in the left ventricle, dividing the left ventricle into two chambers. This may lead to intracardiac haemodynamic abnormalities.
- Although rare, echocardiography can be employed to diagnose and ascertain the existence of intracardiac obstruction.
- The two types of DCLV will be of interest, and the patient’s symptoms and the presence or absence of obstruction will be important indications for surgery.

## Introduction

Double chambered left ventricle (DCLV) is a rare congenital heart defect characterized by the division of the left ventricle (LV) into primary and secondary chambers due to abnormal muscle bundles or septa. Research has shown that abnormal muscle bundles or septa can divide the left ventricle into upper and lower chambers, designated as Type A. In Type A, the primary chamber is located at the base, connected to the aorta and mitral valve, while the secondary chamber is at the apex. If muscle bundles or septa divide the left ventricle into left and right chambers, it is referred to as Type B. In Type B, the primary chamber receives blood from the mitral valve and ejects it into the aortic valve, while the secondary chamber is on the lateral wall.^1–4^ This report presents two case studies, each of which illustrates a different type of DCLV. We analyse the structural characteristics of these two DCLV types using multimodal imaging, providing insights for clinical diagnosis.

## Summary figure

**Figure:**
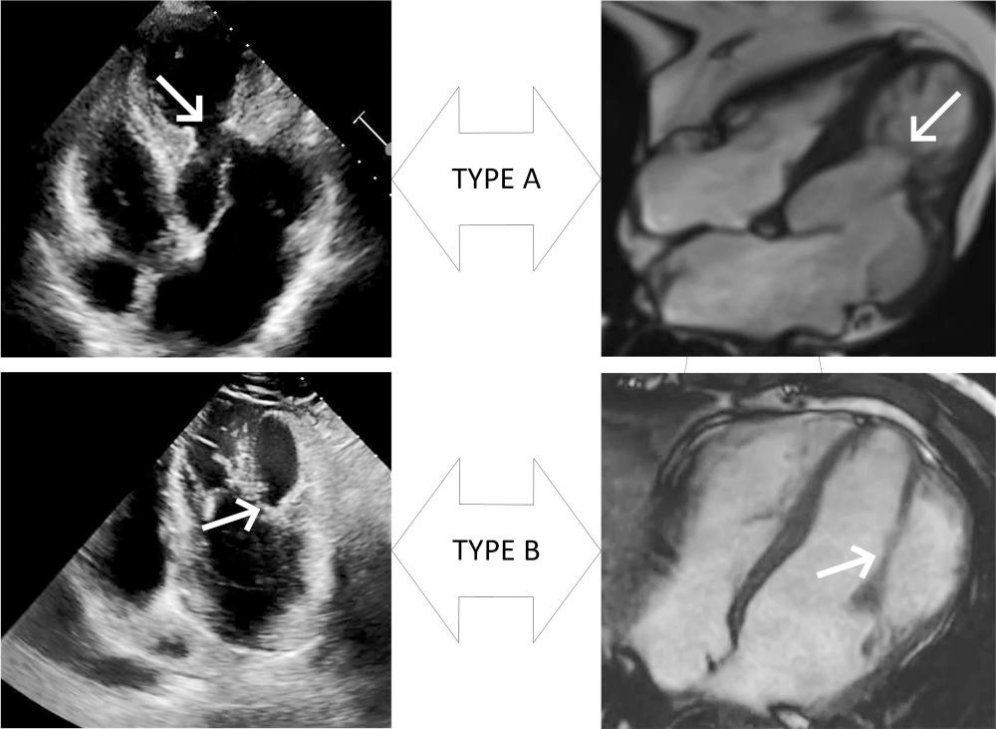


## Patient 1

**Figure 1:**
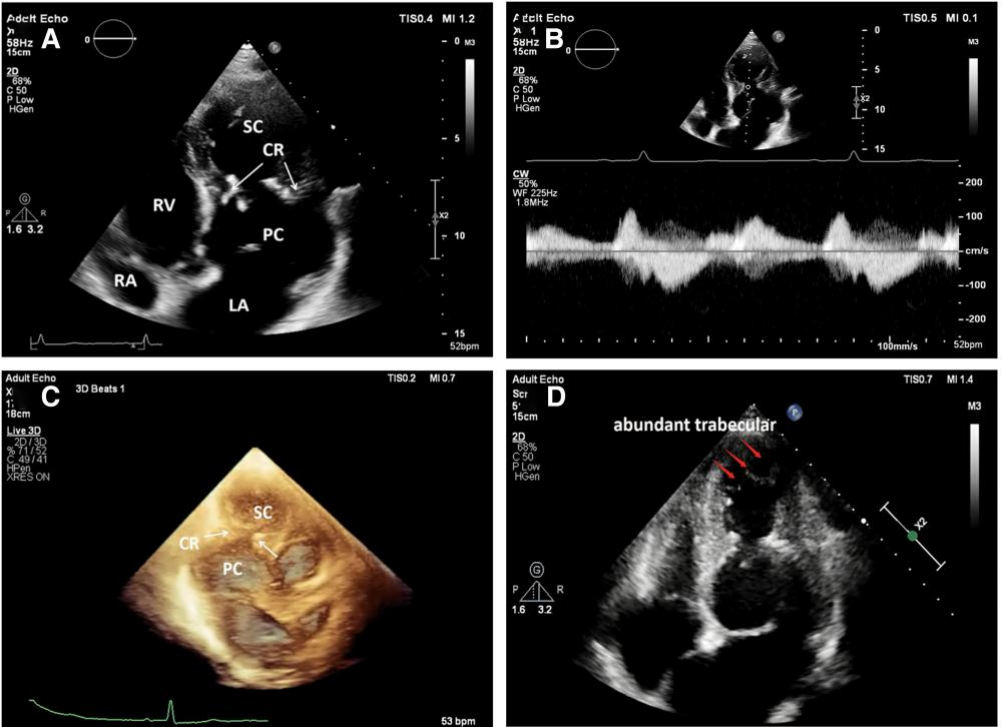
Echocardiographic image of a double chambered left ventricle of type A. *(A)*, two-dimensional transthoracic echocardiography showing apical four-chamber heart and apical two-chamber heart views; *(B)*, spectrum of blood flow at the constriction ring with the cardiac cycle; *(C)*, three-dimensional echocardiography showing apical long-axis views; *(D)*, apical four-chamber view of the heart showing abundant trabecular echogenic structures detected in the apical portion of the left ventricle. LV, left ventricle; PC, the primary chamber; SC, the secondary chamber; LA, left atrium; RA, right atrium; RV, right ventricle; CR, constriction ring.

**Figure 2:**
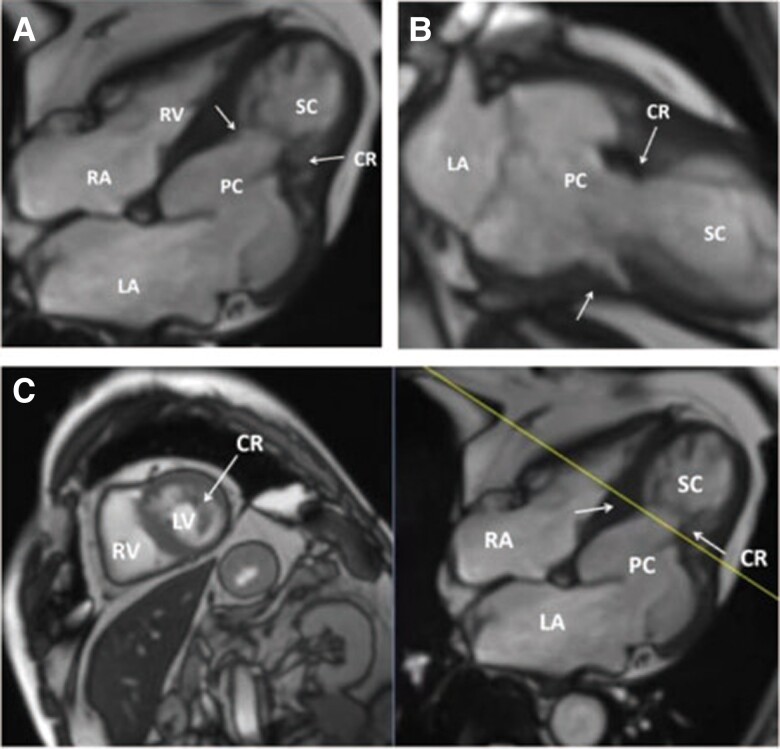
Cardiac magnetic resonance imaging. *(A)*, *(B)*, and *(C)*, Cardiac magnetic resonance showing apical four-chamber heart, apical two-chamber heart, and short-axis views corresponding to dividing muscular shelves.

The initial case involved a 69-year-old male patient who had presented with chest tightness and discomfort for a period of two weeks. At the time of admission, the physical examination and vital signs were found to be within normal parameters during the clinical examination. Two-dimensional transthoracic echocardiography (TTE), as depicted in *Figure 1A*, revealed that the mid-LV was characterised by muscular thickening, with poorly defined papillary muscles and a significantly calcified myocardial bundle. This bundle formed a constricting ring, dividing the LV into two distinct chambers: the primary (basal) and the secondary (apical) chambers. The remaining part of the LV displayed normal myocardial thickness. The primary chamber exhibited reduced myocardial contractility, while the secondary chamber displayed normal contractile function, with a slightly reduced global systolic function (left ventricular ejection fraction estimated at ∼48% using the biplane Simpson’s method). The mitral and aortic valves were located in the primary chamber of the LV. Doppler echocardiography demonstrated bidirectional blood flow during diastole (biphasic flow) and systole (monophasic flow), respectively, through the constricting ring, with turbulent flow observed during the early diastolic phase (*Figure 1B*, Supplementary material online, *Movies S1*–*S4*). 3D imaging echocardiography provided enhanced visualisation of the calcified constriction ring in the mid-LV, revealing increased thickness and hypertrophy of the left ventricular papillary muscles that contributed to its formation (*Figure 1C*, Supplementary material online, *Movies S5*–*S7*). The area of the constriction ring was approximately 1.9 square centimetres, as estimated through 3D tracing. Therefore, the diagnosis of DCLV was confirmed using 2D, 3D, and Doppler echocardiography. Furthermore, abnormal trabecular echoes were observed in the patient’s apical myocardium (*Figure 1D*), suggesting a possible coexistence of excessive myocardial trabecularization in the secondary chamber of the LV. The patient was subsequently subjected to cardiac magnetic resonance imaging, which corroborated the echocardiographic findings. The MRI revealed a protruding myocardial bundle in the mid-LV, further confirming the diagnosis of DCLV (*Figure 2A–C*).

## Patient 2

**Figure 3:**
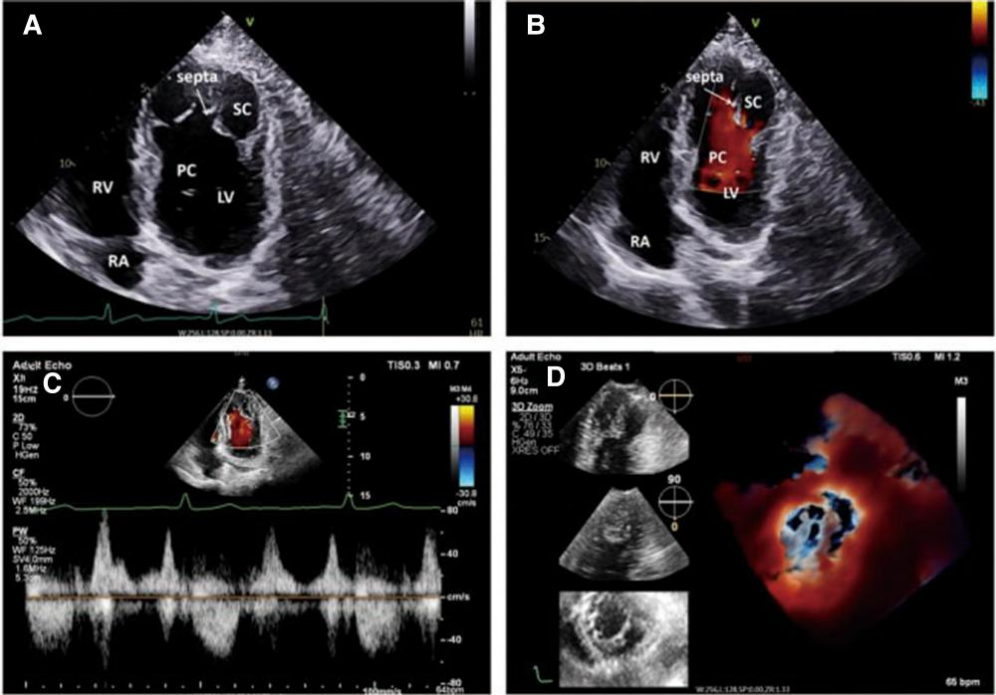
Echocardiographic image of a double chambered left ventricle of type B. *(A)* and *(B)*, two-dimensional transthoracic echocardiography shows the morphology and blood flow in the double chambered left ventricle in a similar apical four-chamber cardiac view; *(C)*, the spectrum of blood flow velocity through the opening of the secondary chamber; *(D)*, three-dimensional echocardiography clearly shows the morphological structure of the double chambered left ventricle.

The second case involves a 48-year-old male patient, incidentally found to have DCLV during a routine physical examination, with no reported discomfort or specific past medical history. The clinical examination was normal and there was no chest pain. *Figure 3A* and *B* from the TTE revealed an abnormal muscle bundle in the lower part of the left ventricular cavity. This bundle, approximately 0.6 cm thick and 3.5 cm long, originated from the apex of the left ventricle and divided the lower portion of the left ventricular cavity into primary and secondary chambers. In this arrangement, the right chamber is the primary chamber, and the left chamber is the secondary chamber (see Supplementary material online, *Movies S8* and *S9*). colour Doppler flow imaging (CDFI) identified blood flow entering and exiting the opening of the secondary chamber with relatively increased velocities. The estimated peak systolic and diastolic velocities were ∼0.54 and 0.64 m/s, respectively, as shown in *Figure 3C*. The primary chamber showed smooth blood flow, and a small amount of low-velocity communicating flow was detected within the muscle bundle separating the two chambers. Cardiac function measurements demonstrated normal left heart function (left ventricular ejection fraction of ∼64.6%). Three-dimensional imaging echocardiography provided a clearer depiction of the internal structure of the left ventricle. It highlighted the complex muscle bundles that divided the left ventricle into differently sized chambers (*Figure 3D*, Supplementary material online, *Movie S10*). Cardiac magnetic resonance imaging revealed more pronounced muscle bundles in the proximal lateral wall of the middle and lower left ventricle, dividing the left ventricle into right and left chambers. Consequently, this patient is classified as having Type B DCLV.

Since neither patient exhibited significant intracardiac haemodynamic abnormalities, no treatment measures were implemented. Regular outpatient follow-up was recommended for monitoring.

## Discussion and conclusion

Both forms of DCLV are clinically uncommon. Patient 1 has been diagnosed for nearly one year and has attended two outpatient follow-ups. Patient 2 has been diagnosed for nearly six months and has attended one outpatient follow-up. Both patients remain in stable overall condition, with no adverse cardiac events reported. DCLV is an exceedingly rare condition in adults, often discovered incidentally during cardiac function assessments due to its asymptomatic nature. Recent imaging advancements have revealed that congenital morphological division abnormalities of the right ventricle are more frequently encountered than those of the left ventricle.^5,6^ The majority of studies on DCLV have been published as case reports, and its pathogenesis remains uncertain. Leung *et al*.^7^ conducted a study that analysed the crucial role of Rac1 in the development of the cardiac septum, suggesting that a deficiency of Rac1 can impair cardiomyocyte elongation and cytoskeleton organization, resulting in a bifid cardiac apex. It has also been proposed that DCLV may arise from endocardial elastofibrillar hyperplasia and specific cardiomyopathies that impair the contraction of the primary chamber of the LV.^8^ In this case, alongside DCLV, the patient presents signs of left ventricular non-compaction, raising the possibility of an alternative mechanism. Inadequate myocardial compaction tends to manifest as excessive trabecularization at the apex, and the septal shelf in the left ventricle could potentially result from an abnormal fusion of trabecular components during development.^9^ While the exact involvement of abnormal trabecularized myocardium in the formation of the septal shelf remains unknown, it is clear that papillary muscle formation in both ventricles is closely linked to this process.^10^ The exact mechanism of DCLV is still unknown. Patients are advised to have regular outpatient follow-ups and echocardiograms, focusing on the left ventricular ejection fraction. Early administration of cardioprotective drugs may benefit patients if a reduced ejection fraction is detected.

## Supplementary Material

ytaf023_Supplementary_Data

## Data Availability

The data underlying this article will be shared on reasonable request to the corresponding author. No data were generated or analysed during the study.

## Lead author biography

Dr Fang Aijuan is a physician with a master’s degree in medicine. She is currently employed at the Drum Tower Hospital of Nanjing University School of Medicine in China, where she performs diagnostic work involving echocardiography. She has been employed in this capacity for over ten years. She exhibits a kind and responsible attitude towards her patients and has consistently demonstrated enthusiasm for her clinical work.

## Notes

**Consent:** The patient consented to the publication of this case in compliance with COPE guidelines.

**Funding:** This research was funded by the National Natural Science Foundation of China (No. 81571691).
